# Differences in Clinical and Dietary Characteristics, Serum Adipokine Levels, and Metabolomic Profiles between Early- and Late-Onset Gout

**DOI:** 10.3390/metabo11060399

**Published:** 2021-06-18

**Authors:** Young Sun Suh, Hae Sook Noh, Hyun-Jin Kim, Yun-Hong Cheon, Mingyo Kim, Hanna Lee, Hyun-Ok Kim, Sang-Il Lee

**Affiliations:** 1 Division of Rheumatology, Department of Internal Medicine, Gyeongsang National University Changwon Hospital, Changwon 51472, Korea; tatabox123@hanmail.net; 2 Division of Rheumatology, Department of Internal Medicine, Gyeongsang National University College of Medicine, Jinju 52727, Korea; haesooknoh@hanmail.net (H.S.N.); hong369c@naver.com (Y.-H.C.); mingyokim1@gmail.com (M.K.); hanna890117@gmail.com (H.L.); 3 Department of Internal Medicine, Institute of Health Science, Gyeongsang National University Hospital, Jinju 52727, Korea; 4 Division of Applied Life Sciences (BK21 plus), Department of Food Science & Technology, and Institute of Agriculture and Life Science, Gyeongsang National University, Jinju 52828, Korea; hyunjkim@gnu.ac.kr; 5 Division of Rheumatology, Department of Internal Medicine, Gyeongsang National University Hospital, Jinju 52727, Korea

**Keywords:** gout, age of onset, adipokines, lipid metabolism

## Abstract

This study aimed to identify differences in clinical and dietary characteristics, serum adipokine levels, and metabolomic profiles between early- and late-onset gout. Eighty-three men with gout were divided into an early-onset group (*n* = 38, aged < 40 years) and a late-onset group (*n* = 45, aged ≥ 40 years). Dietary and clinical information was obtained at baseline. Serum adipokines, including adiponectin, resistin, leptin, and plasminogen activator inhibitor-1 (PAI-1), were quantified by a Luminex multiplex immunoassay. Metabolite expression levels in plasma were measured in 22 representative samples using metabolomics analysis based on ultra-performance liquid chromatography coupled with quadrupole time-of-flight mass spectrometry. Average body mass index, rate of consumption of sugar-sweetened beverages, and serum uric acid levels were significantly higher in the early-onset group (*p* < 0.05), as was the PAI-I concentration (105.01 ± 42.45 ng/mL vs. 83.76 ± 31.16 ng/mL, *p* = 0.013). Changes in levels of metabolites mostly involved those related to lipid metabolism. In the early-onset group, acylcarnitine analog and propylparaben levels were downregulated and negatively correlated with the PAI-1 concentration whereas LPC (22:6) and LPC (18:0) levels were upregulated and positively correlated with the PAI-1 concentration. Dietary and clinical features, serum adipokine concentrations, and metabolites differed according to whether the gout is early-onset or late-onset. The mechanisms of gout may differ between these groups and require different treatment approaches.

## 1. Introduction

Gout is the most common type of inflammatory arthritis in adults and is closely associated with hypertension, insulin resistance, obesity, cardiovascular disease, and other metabolic diseases [1,2]. Gout is traditionally recognized to be a disease affecting middle-aged men. However, the prevalence of gout in adults under 40 years of age has been increasing in recent years [3]. With the exception of genetic factors, the leading cause of the increasing prevalence of early-onset gout is thought to be obesity due to changes in dietary habits, in particular fructose intake [4,5]. Consumption of large amounts of sugar-sweetened beverages (SSBs) and dietary fructose is associated with metabolic disease and gout [6,7]. However, the underlying mechanisms are largely unknown.

Given the recent increase in the number of patients with early-onset gout, several studies have investigated the characteristics of gout according to age of onset. One recent study found that patients with early-onset gout were more likely to have polyarticular flares, higher serum uric acid levels, and higher rates of metabolic syndrome [8]. In a cross-sectional observational study, patients with onset of gout before the age of 40 years had more frequent flares, more joints clinically affected, and fewer cardiovascular, cerebrovascular, and renal comorbidities at presentation but were at higher risk of cardiovascular events [9]. Furthermore, a stratified analysis conducted as part of a meta-analysis of cohort studies revealed a gradual increase in the risk of myocardial infarction with younger age of gout onset [10].

Therefore, there are differences in the clinical features between early-onset and late-onset gout, suggesting that the mechanisms of gout may be different between these two groups. However, there are still no studies of the mechanisms involved in early-onset gout. We hypothesized that there would be a difference in expression levels of serum adipokines and metabolites according to whether gout was early-onset or late-onset and that this difference might be related to the difference in clinical features. Therefore, the aims of this study were to compare the dietary and clinical characteristics of patients with early-onset gout with those of patients with late-onset gout and to determine whether there are differences in serum adipokine and metabolite expression levels between these two groups.

## 2. Results

### 2.1. Clinical Features in the Early-Onset and Late-Onset Groups

There were 38 patients (45.8%) in the early-onset group and 45 (54.2%) in the late-onset group. The median age was 34.5 years (29.5–39.3) in the early-onset group and 54.0 years (49.5–59.0) in the late-onset group. The median age of onset was 29.5 years (23.8–34.0) in the early-onset group and 50.0 years (47.0–54.0) in the late-onset group. The mean BMI was significantly higher in the early-onset group (28.0 vs. 24.8, *p* < 0.001), as was the SSB consumption rate (78.4% vs. 38.6%, *p* < 0.001) and the mean serum uric acid level (8.3 mg/dL vs. 7.3 mg/dL, *p* = 0.010). The estimated glomerular filtration rate was higher in the early-onset group than in the late-onset group but was within the clinically normal range in both groups. There was no significant between-group difference in disease duration, comorbidities, alcohol consumption rate, or serum lipid profile (Table 1).

### 2.2. Serum Adipokines in the Early-Onset and Late-Onset Groups

**Figure 1:**
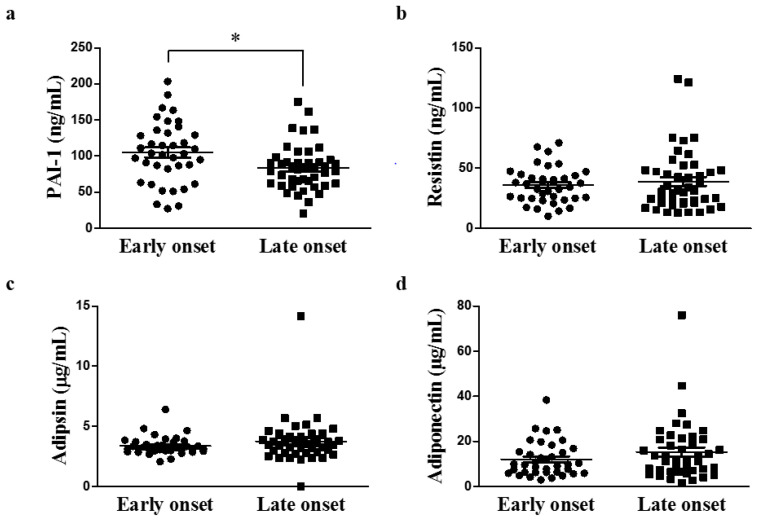
Comparison of serum adipokine concentrations according to age of onset of gout (early-onset group, *n* = 38; late-onset group, *n* = 45; * *p* < 0.05). (**a**) PAI-1 (**b**) Resistin (**c**) Adipsin (**d**) Adiponectin. PAI-1, plasminogen activator inhibitor-1.

The serum PAI-I concentration was significantly higher in the early-onset group than in the late-onset group (105.01 ± 42.45 ng/mL vs 83.76 ± 31.16 ng/mL, *p* = 0.013; Figure 1). The metabolite analysis was performed by randomly selecting 11 patients from each study group. There was a significant difference in the PAI-1 level between the two subgroups (Supplementary Figure S1). Concentrations of other serum adipokines (adiponectin, resistin, and adipsin) were not significantly different between the two groups. This finding sugested that an increased serum PAI-1 concentration may be associated with certain clinical characteristics, such as high BMI, in patients with early-onset gout.

### 2.3. Potential Pathophysiologic Mechanism and Markers of Differences between the Early-Onset and Late-Onset Groups

**Figure 2:**
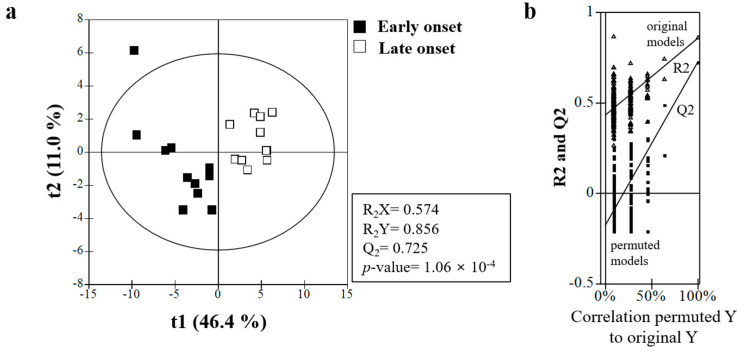
(**a**) Partial least-squares discriminant analysis (PLS-DA) scores obtained from UPLC-Q-TOF MS data. Outliers from the elliptical region of the 95% confidence interval were excluded by Hotelling’s T2 test. The scores plots showed significant separation between the samples based on the parameters of model quality: R2X, R2Y, and Q2Y. (**b**) The PLS-DA model was validated by a permutation test (*n* = 200): *p*-values and intercepts of R2 (Ri) and Q2 (Qi). UPLC−Q-TOF-MS, ultra-performance liquid chromatography coupled with quadrupole time-of-flight mass spectrometry.

The representative sample in this metabolomics study consisted of 22 male patients (early-onset group, *n* = 11; late-onset group, *n* = 11). Various metabolites were identified in serum samples by UPLC-TOF-MS (Supplementary Figure S2). Nuclear magnetic resonance spectrum data were analyzed by principal component analysis and PLS-DA to observe clustering trends of samples obtained from the patients in the two groups. The PLS-DA scores plot (R_2_X = 0.574, R_2_Y = 0.856, Q_2_ = 0.538, *p* = 1.06 × 10^−4^) showed clear separation between the two groups (Figure 2a), and the PLS-DA model was validated by a permutation test (Figure 2b).

**Figure 3:**
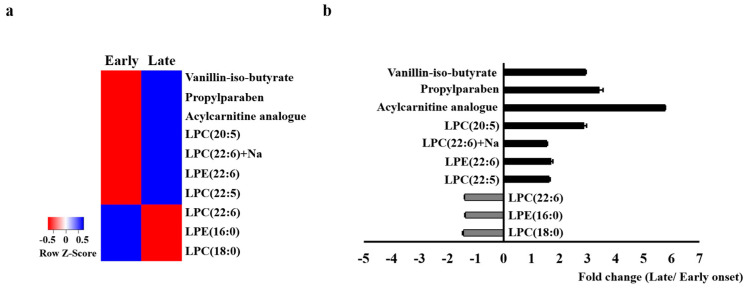
Heatmap of the identified serum metabolites (**a**) and the fold changes (**b**) between the early-onset group and the late-onset group. The color of each section corresponds to the concentration value for each metabolite calculated by the peak area normalization method (red, upregulated; blue, downregulated).

There were significant between-group differences in 10 of the analyzed metabolites (Supplementary Table S3). The heatmap for these metabolites showed remarkable between-group differences in the metabolic profile. In the early-onset group, vanillin-iso-butyrate, propylparaben, acycarnitine analog, lysophosphatidylcholine (LPC) (20:5), LPC (22:6) + Na, LPE (22:6), and LPC (22:5) were downregulated while LPC (22:6), LPE (16:0), and LPC (18:0) were upregulated (Figure 3).

**Figure 4:**
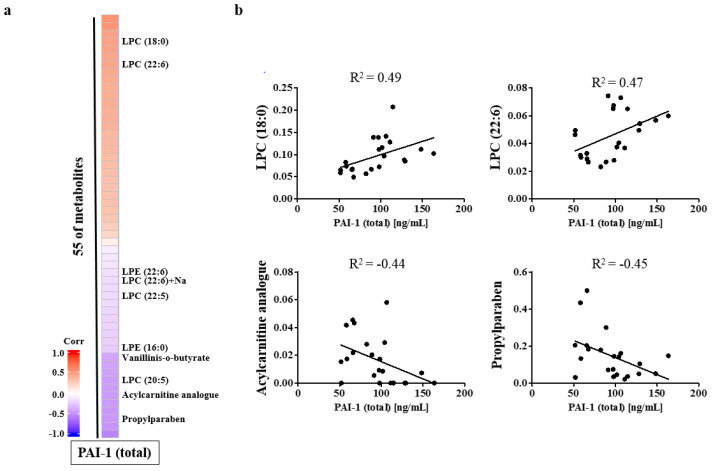
Heatmap for the identified serum metabolites (**a**) and correlation between the serum PAI-1 concentration and each metabolite (**b**). LPC, lysophosphatidylcholine; PAI-1, plasminogen activator inhibitor-1.

There were correlations between the levels of these metabolites and the serum PAI-1 concentration. Acylcarnitine analog and propylparaben levels were negatively correlated with the PAI-1 concentration and the LPC (18:0), and LPC (22:6) levels were correlated positively with the PAI-1 concentration (Figure 4).

## 3. Discussion

In this study, we identified differences in diet and clinical characteristics between patients with early-onset gout and those with late-onset gout. The mean SSB consumption rate and mean BMI were significantly higher in the early-onset group. Moreover, in the early-onset group, the serum PAI-1, LPC (18:0), and LPC (22:6) values were higher and there was a positive correlation between PAI-1 and LPC. The majority of the metabolic alterations involved lipid metabolism. In the early-onset group, it is likely that excessive intake of fructose induced obesity, which led to differences in serum adipokine and metabolite levels.

Our study also found a difference in the clinical features of early-onset and late-onset gout. Recent genomic-wide association studies conducted to determine the cause of these clinical differences have shown that some polymorphisms in ATP-binding cassette G2 (ABCG2) lead to hyperuricemia through renal overload and renal underexcretion and also increase the risk of early-onset gout. The single nucleotide polymorphism ABCG2 rs2231142 is strongly associated with early-onset gout in Polynesian [11], Japanese [12], Czech [13], and other European populations. Although a genetic analysis was not performed in this study, ABCG2 polymorphisms are likely to have affected the age of onset of gout. Further investigations are needed to identify genetic differences among the participants in this study.

The association between fructose and metabolic diseases, including obesity, has been reported previously [14,15]. Several trials have provided consistent evidence for the body weight-increasing effect of fructose [16,17]. In the present study, the SSB intake rate and BMI were higher in the early-onset group and were thought to be related. A recent metabolomics study showed that a high-fructose diet resulted in an increase in the mean LPC levels in obese individuals [18]. Therefore, excessive fructose consumption may significantly affect lipid metabolism.

PAI-1 inhibits plasminogen activators that convert plasminogen to plasmin. Plasmin activates tissue matrix metalloproteinases, which cause degradation of the extracellular matrix in the arterial wall. Elevated levels of PAI- 1 ultimately decrease plasmin formation and may lead to accumulation of extracellular matrix and atherosclerosis [19]. Elevated plasma PAI-1 levels have been associated with impaired fibrinolytic activity in cardiovascular disease [20]. A recent meta-analysis demonstrated that elevated plasma PAI-1 levels are associated with major adverse cardiovascular events [21]. In obesity, PAI-1 expression is markedly increased in adipose tissue, indicating that adipose tissue is a major source of elevated levels of circulating PAI-1 [22]. In this study, patients with early-onset gout were more obese and had higher serum PAI-1 levels than their counterparts with late-onset gout, suggesting that obesity may increase the risk of cardiovascular disease in patients with early-onset gout.

LPC is the main component of oxidized low-density lipoprotein (oxLDL). In recent years, the importance of LPCs in the development of endothelial dysfunction and arteriosclerosis has been highlighted by the finding of high concentrations of LPCs within arteriosclerotic plaques [23,24]. Several metabolomics studies have suggested that specific LPCs may be associated with cardiovascular disease, but the association is not clear. In one study in humans, the levels of some LPC species, including LPC (16:0), LPC (18:0), and LPC (18:1), were approximately two times higher in atherosclerotic plaques associated with symptoms than those in asymptomatic plaques [25]. Another study found that LPC 16:0, LPC 18:1, and LPC 20:4 activated endothelial cells and promoted the release of inflammatory cytokines and apoptosis, which accelerates the development of atherosclerosis [26]. In another study, LPC 20:4 and LPC 22:6 were found to serve as anti-inflammatory lipid mediators and inhibit the inflammation induced by saturated LPC [27]. In our present study, elevations in LPC (18:0) and LPC (22:6) were prominent in patients with early-onset gout. Further research is needed to determine which specific LPC(s) could be associated with cardiovascular disease in patients with early-onset gout.

A previous study demonstrated that the uptake of oxLDL and its lipid component LPC into adipocytes triggered aberrant reactive oxygen species-mediated PAI-1 expression, which may be involved in the pathogenesis of metabolic syndrome and atherosclerosis [28]. Another study showed that LPC stimulates the enzyme activity of PAI-1 and suppresses that of tissue plasminogen activator [29]. In this study, we found elevated PAI-1 and LPC and a statistically significant positive correlation between serum PAI-1 and LPC in patients with early-onset gout. Considering the above findings, the elevation of PAI-1 and LPC and their positive correlation may be related to an increased risk of cardiovascular disease in patients with early-onset gout.

This study had several limitations. First, it was conducted at a single center and had a small sample size. Larger multicenter controlled studies are needed to confirm and extend the findings of this investigation. Second, in order to minimize the effects of drugs that may be involved in metabolism, the study was conducted only in newly diagnosed patients. However, the possibility that confounding may have influenced the results cannot be excluded.

## 4. Materials and Methods

### 4.1. Study Population

The study population comprised 83 male patients with a diagnosis of gout who attended the Gyeongsang National University Hospital between January 2018 and December 2019.The participants were limited to patients with a new diagnosis of gout; thus, this study was conducted using serum collected before patients received urate-lowering and lipid-lowering drugs. Gout was defined by the American College of Rheumatology/European League against Rheumatism classification criteria [30]. Cases of gout that were drug-induced, secondary to chronic kidney disease or uromodulin-related diseases, or associated with a family or individual history of purine enzymopathy (hypoxanthine-guanine phosphoribosyl transferase deficiency, or phosphoribosyl pyrophosphate synthetase superactivity), were excluded. The patients were divided into an early-onset group (aged < 40 years) and a late-onset group (aged ≥ 40 years) [9]. Dietary and clinical information, including age, age at onset, disease duration, comorbidities, body mass index (BMI; calculated as kg/m^2^), and consumption of SSBs and alcohol, was obtained at baseline. The results of laboratory investigations were examined at baseline. Serum extracted from fasting blood samples was stored immediately at −70 °C until measurement of serum uric acid, glucose, creatinine, total cholesterol, triglycerides, high-density lipoprotein, low-density lipoprotein, serum adipokine, and metabolite levels.

The study was approved by the Institutional Review Board of Gyeongsang National University Hospital (approval number GNUH2014-02-013). Informed consent was obtained from all study participants.

### 4.2. Serum Adipokine Assay

Serum adipokines were quantified by the Luminex multiplex immunoassay using Human Adipokine Magnetic Bead Panel 1 kits (HADK1MAG-61 K; Millipore, St. Charles, MO, USA) on a Luminex 200 analyzer (Luminex Corporation, Austin, TX, USA) according to the manufacturers’ instructions. The panel consisted of adiponectin, resistin, leptin, and plasminogen activator inhibitor type 1 (PAI-1). Analyses were performed in duplicate, and serial dilutions were performed according to the detection range of each panel.

### 4.3. Identification of Serum Metabolites by UPLC-Q-TOF-MS

Plasma metabolomics analysis based on ultra-performance liquid chromatography coupled with quadrupole time-of-flight mass spectrometry (UPLC-Q-TOF-MS) was used to identify differentially expressed metabolites in patients with early-onset gout and those with late-onset gout.

### 4.4. Chemicals and Reagents

Water (LC-MS grade), acetonitrile (LC-MS grade ≥ 99.9%), methanol (LC-MS grade ≥ 99.9%), formic acid (≥98%), acetone (LC-MS grade ≥ 99.9%), and terfenadine were purchased from Merck (Darmstadt, Germany). 

### 4.5. Sample Preparation

Serum samples were precipitated by the addition of cold acetone containing terfenadine as an internal standard. After centrifugation, the dried supernatant was dissolved in 20% aqueous methanol.

### 4.6. Metabolic Profiling of Serum Samples by UPLC-Q-TOF-MS

One microliter of the eluted sample was injected into a Waters Acquity UPLC-Q-TOF-MS system (Waters, Milford, MA, USA) equipped with an Acquity UPLC BEH C18 column (2.1 × 100 mm, 1.7 μm; Waters). The mobile phase consisted of solvent A (0.1%) and solvent B (acetonitrile containing 0.1% formic acid). Analytes was eluted in a gradient of acetonitrile containing 0.1% formic acid at a flow rate of 0.35 mL/min for 15 min; eluted metabolites were detected by Q-TOF-MS connected to the UPLC system. The Q-TOF-MS was operated in electrospray ionization (ESI)-positive mode. The voltages of the capillary and sampling cone were set at 3 kV and 40 V, respectively. Other conditions were optimized as follows: desolvation gas flow rates, 800 L/h; temperature, 400 °C; ion source temperature, 100 °C. Leucine-enkephalin (*m*/*z* 556.2771 in ESI-positive mode) was used as the lock mass for all analyses to ensure accuracy and reproducibility.

### 4.7. Data Processing

All Q-TOF MS data were analyzed using UNIFI software version 1.8 (Waters) including retention times, *m*/*z*, and ion intensities. Metabolites were searched for in the Human metabolome database (http://www.hmdb.ca, 1 January 2020), Chemspider database (www.chemspider.com, 1 January 2020), and Metlin database (www.metlin.scripps.edu, 1 January 2020), and/or confirmed by standard samples based on both retention times and mass spectra.

### 4.8. Statistical Analysis

Continuous variables are summarized as the median and interquartile range and categorical variables as the frequency count and percentage. Patient characteristics were compared between the early-onset and late-onset groups using the chi-square statistic for categorical variables and the Wilcoxon rank-sum statistic for continuous variables. The aligned and normalized LC/MS datasets were examined using SIMCA-P+ version 12.0.1 (Umetrics, Umea, Sweden) for multivariate statistical analysis as described previously [31], principal component analysis, partial least-squares discriminant analysis (PLS-DA), heat map visualization, and correlation coefficient analysis. Multiple comparisons among groups were performed by one-way analysis of variance with Duncan’s test using SPSS version 24.0 (IBM Corp., Armonk, NY, USA). Results are expressed as the mean ± the standard error of the mean. Statistical significance was set at *p* < 0.05.

## 5. Conclusions

This is the first study to find differences in dietary and clinical characteristics, serum adipokine levels, and the metabolic profile between patients with early-onset gout and those with late-onset gout. Compared with patients who had late-onset gout, patients with early-onset gout had a higher body mass index and increased LPC and serum PAI-1 values; furthermore, there was a correlation between the LPC and PAI-1 values. Given that an elevated serum PAI-1 concentration is associated with cardiovascular disease, there may be an increased risk of cardiovascular disease in patients with early-onset gout. Our study findings indicate that different approaches, including a more aggressive medical treatment strategy, may be required in patients with early-onset gout.

## Figures and Tables

**Table 1:** Comparison of clinical features between the early-onset and late-onset gout groups.

Variables	Early-Onset Group (*n* = 38)	Late-Onset Group (*n* = 45)	*p*-Value
Age (years)	34.5 (29.5–39.3)	54.0 (49.5–59.0)	<0.001
Age at onset (years)	29.5 (23.8–34.0)	50.0 (47.0–54.0)	<0.001
Disease duration (months)	42.0 (24.0–108.0)	48.0 (9.0–90.0)	0.407
Height (cm)	172.5 (170.0–177.3)	170.0 (166.5–173.0)	0.004
Body weight (kg)	83.5 (74.8–90.8)	72.0 (65.0–79.0)	<0.001
Body mass index (kg/m^2^)	28.0 (25.4–29.8)	24.8 (23.5–25.9)	<0.001
Comorbidity (yes, %)	22 (57.9)	33 (73.3)	0.138
Tophi (yes, %)	9 (23.7)	11 (24.4)	0.936
Alcohol (yes, %)	32 (84.2)	38 (84.4)	0.977
SSB consumption (yes, %)	29 (78.4)	17 (38.6)	<0.001
No	8 (21.6)	27 (61.4)	
1–2/month	11 (29.7)	4 (9.1)	
1–2/week	7 (18.9)	2 (4.5)	
3–4/week	5 (13.5)	1 (2.3)	
5–6/week	2 (5.4)	7 (15.9)	
Every day	4 (10.8)	3 (6.8)	
Laboratory data			
Uric acid (mg/dL) (3.5–7.2)	8.3 (6.5–9.6)	7.3 (5.8–8.0)	0.010
Fasting glucose (mg/dL) (74–106)	98.5 (92.0–117.5)	107.5 (98.0–127.5)	0.265
Creatinine (mg/dL) (0.67–1.17)	0.91 (0.85–1.12)	1.00 (0.86–1.20)	0.136
eGFR (MDRD)	108.5 (87.5–115.8)	93.0 (66.5–101.8)	0.007
AST (IU/L) (1–37)	23.5 (17.0–32.5)	22.0 (18.0–28.8)	0.432
ALT (IU/L) (0–41)	30.0 (24.0–51.0)	27.0 (17.0–32.0)	0.338
Total cholesterol (mg/dL)(120–200)	197.0 (179.5–227.5)	183.5 (157.8–205.5)	0.055
Triglycerides (mg/dL) (0–150)	155.0 (112.5–389.0)	131.0 (90.0–246.3)	0.304
HDL cholesterol (mg/dL) (40–60)	43.0 (35.5–55.5)	42.0 (38.8–60.5)	0.708
LDL cholesterol (mg/dL) (0–130)	146.0 (91.5–162.5)	123.0 (98.5–145.0)	0.363
C-reactive protein (mg/L) (0.0–5.0)	0.6 (0.1–1.0)	0.3 (0.1–1.5)	0.634

Continuous variables are shown as the median (interquartile range) and categorical variables as the number (percentage). ALT, alanine aminotransferase; AST, aspartate amniotransferase; eGFR, estimated glomerular filtration rate; LDL, low-density lipoprotein; HDL, high-density lipoprotein; MDRD, Modification of Diet in Renal Disease; SSB, sugar-sweetened beverages.

## Data Availability

The data presented in this study are contained within the article or supplementary material.

## Supplementary Materials

The following are available online at https://www.mdpi.com/article/10.3390/metabo11060399/s1, Figure S1: Comparison of PAI-1 concentrations according to age of onset, Figure S2: Base peak intensity chromatograms of plasma metabolic profiles in a serum sample based on ultra-performance liquid chromatography coupled with quadrupole time-of-flight mass spectrometry analysis, Table S3: Identification of major metabolites contributing to separation among the sample groups.
