# Supplementary material for: Differences in Clinical and Dietary Characteristics, Serum Adipokine Levels, and Metabolomic Profiles between Early- and Late-Onset Gout
Source: Metabolites. 2021 Jun 18;11(6):399. doi: 10.3390/metabo11060399 (PMC8234189; doi:10.3390/metabo11060399)
Supplement: Supplementary file 1 [file metabolites-11-00399-s001.zip › metabolites-1245417-supple-proofed/Supplementary Files/supplementary materials.pdf]

## SUPPLEMENTARY MATERIALS

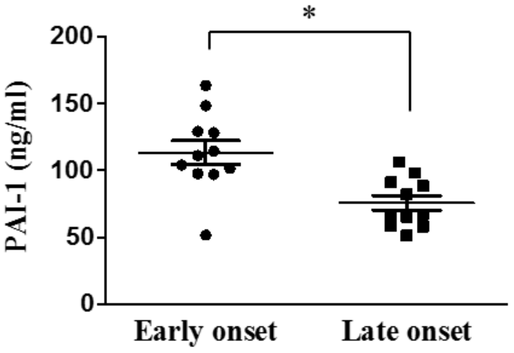


**Figure S1.** Comparison of PAI-1 concentrations according to age of onset (n=11 in each group, *p<0.005)

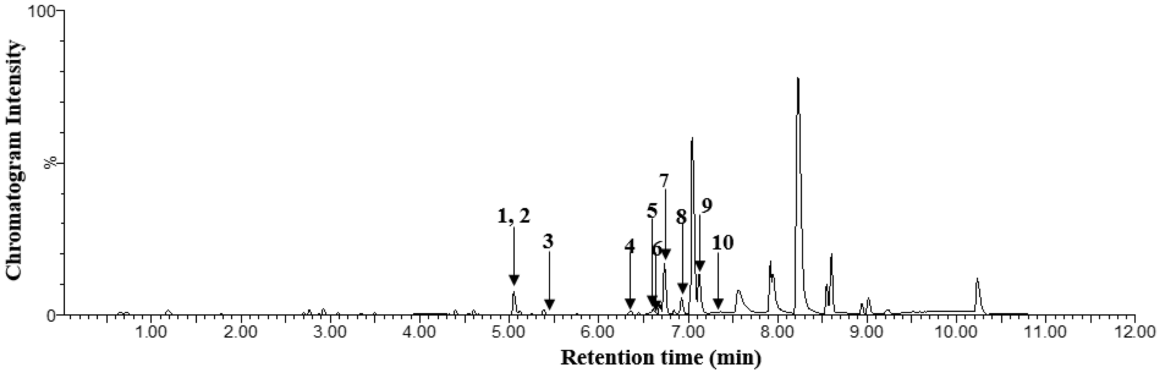


**Figure S2**. Base peak intensity chromatograms of plasma metabolic profiles in a serum sample based on ultra-performance liquid chromatography coupled with quadrupole time-of-flight mass spectrometry analysis. 1, vanillin-iso-butyrate; 2, propylparaben/4-propoxybenzoic acid; 3, acylcarnitine analog/[2-(dioctylamino)-2-oxoethoxy]acetic acid; 4, LPC (20:5); 5, LPC (22:6)+Na; 6, LPE (22:6); 7, LPC (22:5); 8, LPC (22:6); 9, LPE (16:0); 10. LPC (18:0)

**Table S3.** Identification of major metabolites contributing to separation among the sample groups.

| No. | RT (min) | Compound | Exact mass (M+H) | MS Fragments | VIP | *p*-value |
|---|---|---|---|---|---|---|
| 1 | 5.03 | vanillinis-iso-butyrate | 223.0948 | 181 | 1.05 | 0.001 |
| 2 | 5.04 | Propylparaben / 4-propoxybenzoic acid | 181.0846 | 163 | 1.08 | 0.002 |
| 3 | 5.45 | Acylcarnitine analogue / [2-(Dioctylamino)-2-oxoethoxy]acetic acid | 358.2934 | 282 | 1.13 | 0.001 |
| 4 | 6.34 | LPC(20:5) | 542.3241 | 184, 104 | 1.27 | 0.000 |
| 5 | 6.59 | LPC(22:6)+Na | 590.3211 | 385, 550 | 0.85 | 0.013 |
| 6 | 6.62 | LPE(22:6) | 526.2926 | 385 | 0.89 | 0.008 |
| 7 | 6.74 | LPC(22:5) | 570.3548 | 104, 387, 552 | 0.73 | 0.042 |
| 8 | 6.89 | LPC(22:6) | 568.3364 | 550, 383 | 0.77 | 0.036 |
| 9 | 7.08 | LPE(16:0) | 454.2917 | 436 | 0.81 | 0.027 |
| 10 | 7.31 | LPC(18:0) | 508.3750 | 184 | 0.78 | 0.030 |
