# Supplementary figures and images for: Differences in Clinical and Dietary Characteristics, Serum Adipokine Levels, and Metabolomic Profiles between Early- and Late-Onset Gout
Source: Metabolites. 2021 Jun 18;11(6):399. doi: 10.3390/metabo11060399 (PMC8234189; doi:10.3390/metabo11060399)

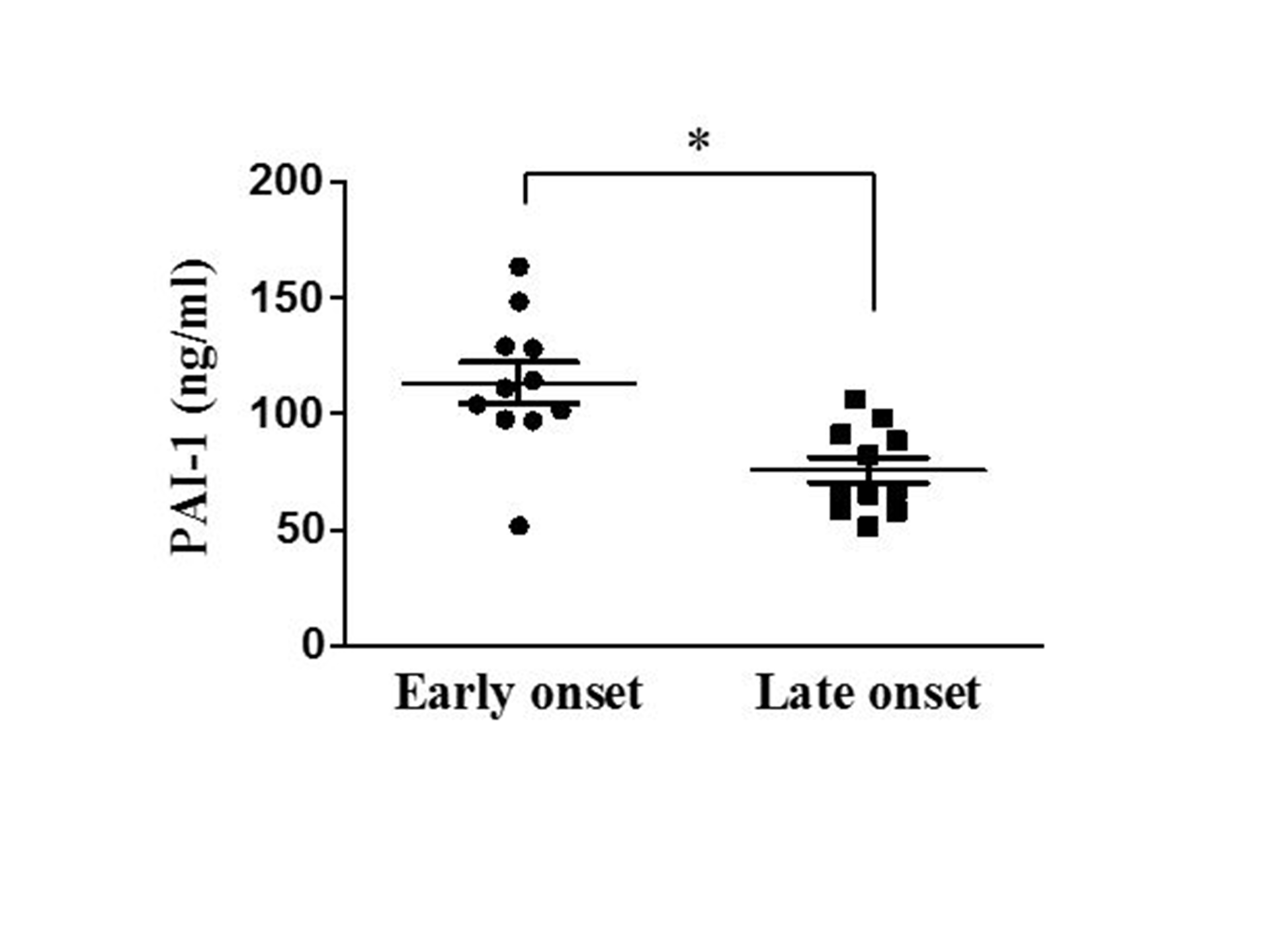

Supplement: Supplementary file 1 [file metabolites-11-00399-s001.zip › metabolites-1245417-supple-proofed/Supplementary Files/Figure S1.tif]

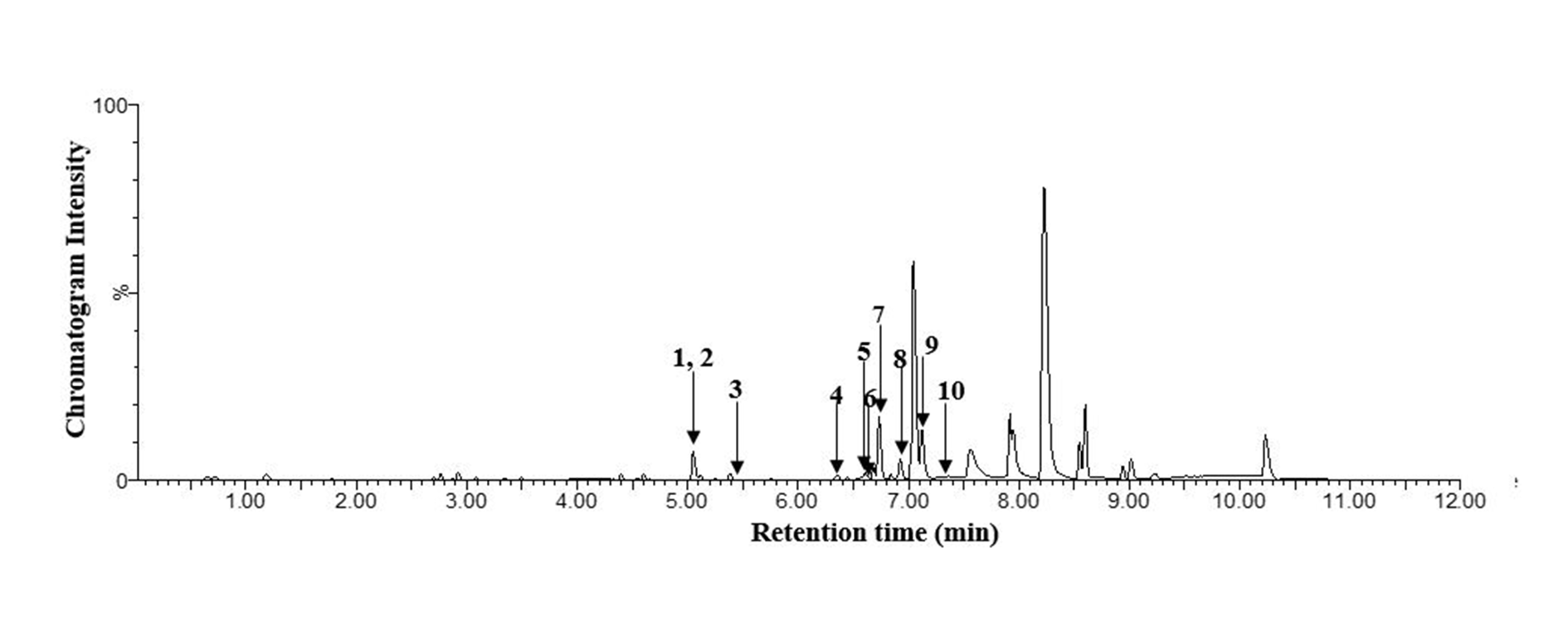

Supplement: Supplementary file 1 [file metabolites-11-00399-s001.zip › metabolites-1245417-supple-proofed/Supplementary Files/Figure S2.tif]

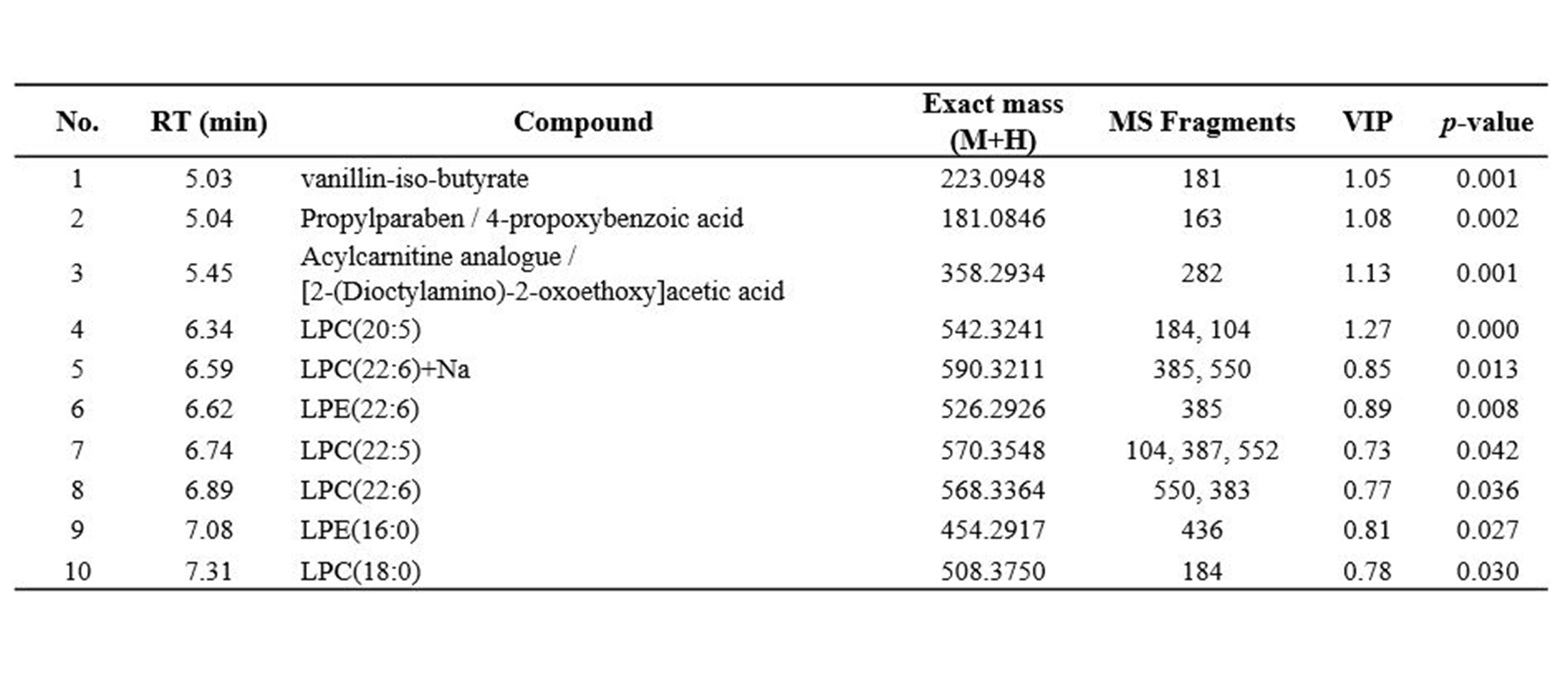

Supplement: Supplementary file 1 [file metabolites-11-00399-s001.zip › metabolites-1245417-supple-proofed/Supplementary Files/Table S3.tif]
